# Lectotypification of five names in the genus *Stellaria* (Caryophyllaceae) in China

**DOI:** 10.3897/phytokeys.170.59527

**Published:** 2020-12-21

**Authors:** Wenqiao Wang, Zhiwei Su, Zhonghui Ma

**Affiliations:** 1 National Demonstration Center for Experimental Plant Science Education, Traditional Chinese Herbal Medicine Resources and Agriculturalization Research Institute, College of Agriculture, Guangxi University, Nanning 530004, China Guangxi University Nanning China; 2 Institute of Marine Drugs, Guangxi University of Chinese Medicine, Nanning 530200, China Guangxi University of Chinese Medicine Nanning China

**Keywords:** Caryophyllaceae, lectotype, *
Stellaria
*

## Abstract

Lectotypification for *Stellaria depressa* Em. Schmid, *S. yunnanensis* Franch., *S. ebracteata* Kom., *S. filicaulis* Makino, and *S. pusilla* Em. Schmid are designated here.

## Introduction

The genus *Stellaria* L. was described by Linnaeus and comprises c. 190 species around the world (Chen and Rabeler 2001; Xu and Ma 2018; Wang et al. 2020; Xu et al. 2020). In China, 69 species were reported, with five new species described recently, of which 33 were endemic (Wu and Ke 1996; Chen and Rabeler 2001; Gan and Li 2014; Xu and Ma 2018; Song et al. 2020; Wang et al. 2020; Yang et al. 2020). During the study on the genus *Stellaria* in China, we found *S. depressa* Em. Schmid, *S. yunnanensis* Franch., *S. ebracteata* Kom., *S. filicaulis* Makino and *S. pusilla* Em. Schmid needed to be lectotypified according to Art. 9.3 and Art. 9.11 of the Shenzhen code (Turland et al. 2018). Hence, these species are lectotypified here after literature survey and specimen examination.

## Materials and methods

Specimens of *Stellaria depressa*, *S. yunnanensis*, *S. ebracteata*, *S. filicaulis* and *S. pusilla* matching the criteria of original material were searched at K, LE, MAK, P, TNS and Z. The lectotype designations in this paper follow the rules of the Shenzhen Code (Turland et al. 2018). All specimens were examined and studied by authors.

## Typification

### Stellaria depressa

Taxon classificationPlantaeCaryophyllalesCaryophyllaceae

Em. Schmid, Repert. Spec. Nov. Regni Veg. 31: 41 (1932)

ABEB1A66-9197-5B1E-99F5-BE6E10CAC831

#### Lectotype

**Figure 1.**
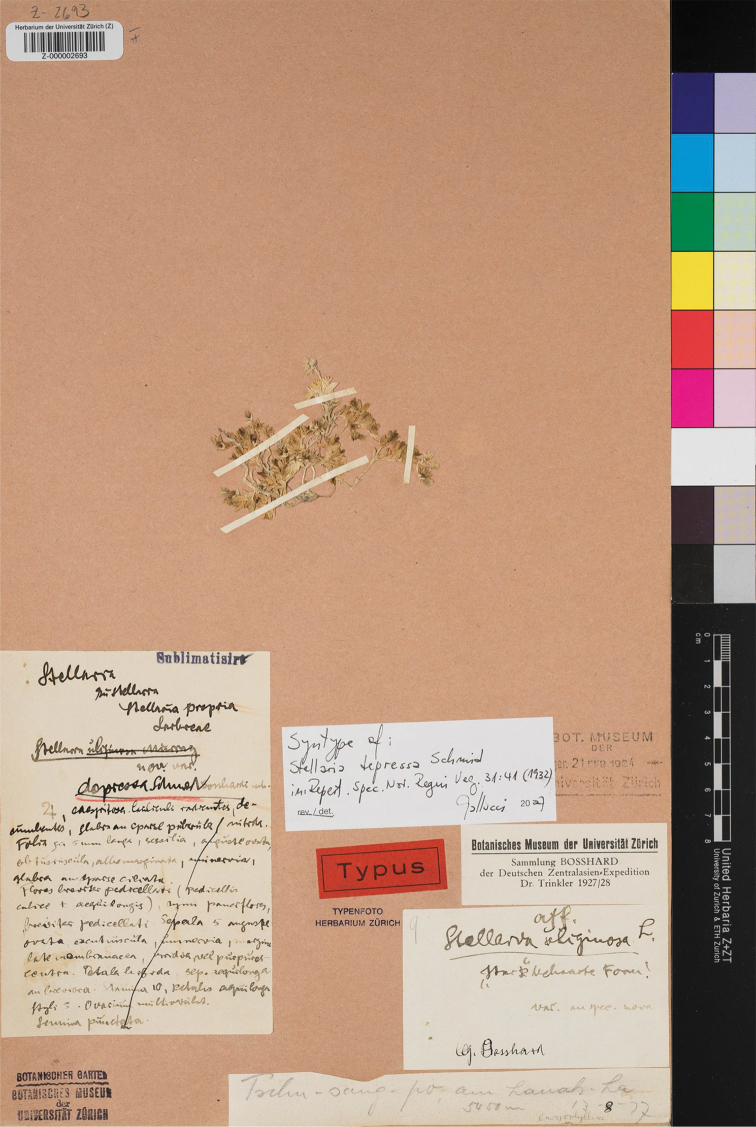
Lectotype of *S. depressa* Em. Schmid (Walter Bosshard, *Bosshard s.n.*, Z000002693).

(designated here):–China, Tschu-sang-po, am Lanak-La, August 13, 1927, *Bosshard s.n.* (Z000002693 digital image!, Figure 1; Isolectotypes: China, Aksai-Chin, *Bosshard s.n.*, Z barcode Z000002691 digital image!, China, Ladakh, Zingrul, *Bosshard s.n.*, Z barcode Z000002692 digital image!).

#### Note.

When Schmid first described *S. depressa*, he cited three specimens “*Bosshard s.n.*, 16. VII. 1927; *Bosshard s.n.*, 13. VIII. 1927; *Bosshard s.n.*, 5. IX. 1927” collected by Bosshard from Ladakh and Tibet, but he didn’t designate any one of them as holotype in the protologue. According to Stafleu and Cowan (1993), Bosshard’s specimens were deposited in W and Z. We traced three specimens of *S. depressa* collected by Bosshard deposited in Z (Z000002693 digital image!, Z000002691 digital image!, Z000002692 digital image!). Although they were collected by Bossard at different time, it seems that they were treated as types since they all have the label “Typus”. However, according to Arts. 9.1, 9.6, and 40 Note 1 of the ICN (Turland et al. 2018), none of them can be treated as holotype, but all should be considered as syntypes. Given a label on the specimen sheet with the description matching the protologue of *S. depressa*, its good preservation, and the perfect presence of flower and inflorescence, Z000002693 is designated here as the lectotype according to Art. 9.3 and 9.4 of the ICN (Turland et al. 2018).

### Stellaria yunnanensis

Taxon classificationPlantaeCaryophyllalesCaryophyllaceae

Franch., Bull. Soc. Bot. France. 33: 433 (1886)

F78B1E5E-D02E-5DE8-8056-FABB80703C90

#### Lectotype

**Figure 2.**
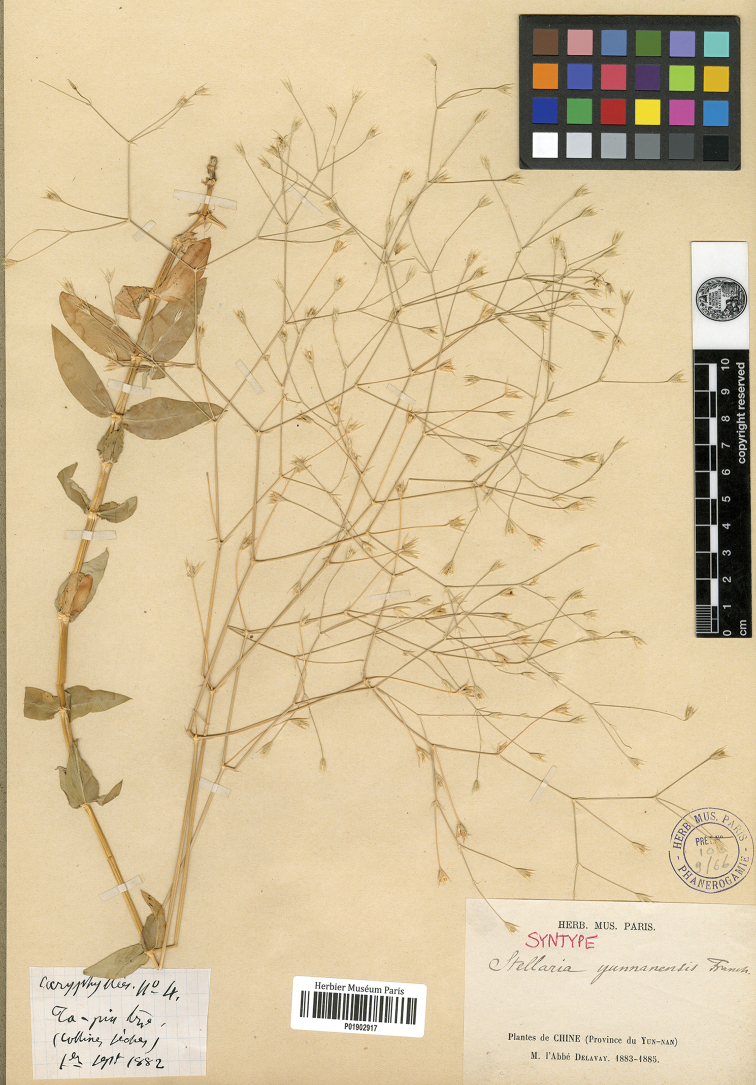
Lectotype of *S. yunnanensis* Franch. (J.M. Delavay, *Delavay 4*, P01902917).

(designated here):–China, Yunnan, Les collines incultes au dessus de Ta pin tze, September 1, 1882, *Delavay 4* (P01902917 digital image!, Figure 2; Isolectotypes: China, Les collines incultes au dessus de Ta pin tze, *Delavay 4*, P barcodes P01902916 and P01902918–P01902919 digital images!, China, Les pâturages au pied du Tsang chan, au dessus de Ta-li, *Delavay 1*, P barcodes P01902913–P01902915 digital images!, China, Da-pin-tze, *Delavay s.n.*, K barcode K000723671 digital image!).

#### Note.

Franch described *S. yunnanensis* based on two specimens “*Delav. Caryoph. n. 1*, 4. jul. 1882; *Delav. Caryoph. n. 4*, 1. sept. 1882” collected by Delavay from Yunnan, China, without designating any one of them as holotype in the protologue. According to Stafleu and Cowan (1976), Delavay’s specimens were deposited in K, P and PC. Eight original materials were found in P (P01902913–P01902919 digital images!) and K (K000723671 digital image!), which all have Delavay’s annotation and are well preserved. The specimens of P all bear the information “Syntype *Stellaria yunnanensis* Franch.”. P01902917 well presents inflorescence and lower part of the plant and is in line with the protologue. So P01902917 is designated here as the lectotype according to Art. 9.3 and 9.4 of the ICN (Turland et al. 2018).

### Stellaria ebracteata

Taxon classificationPlantaeCaryophyllalesCaryophyllaceae

Kom., Trudy Imp. S.-Peterburgsk. Bot. Sada. 18: 441 (1901)

70B720C1-6521-532B-B898-BF14018F1DF1

#### Lectotype

**Figure 3.**
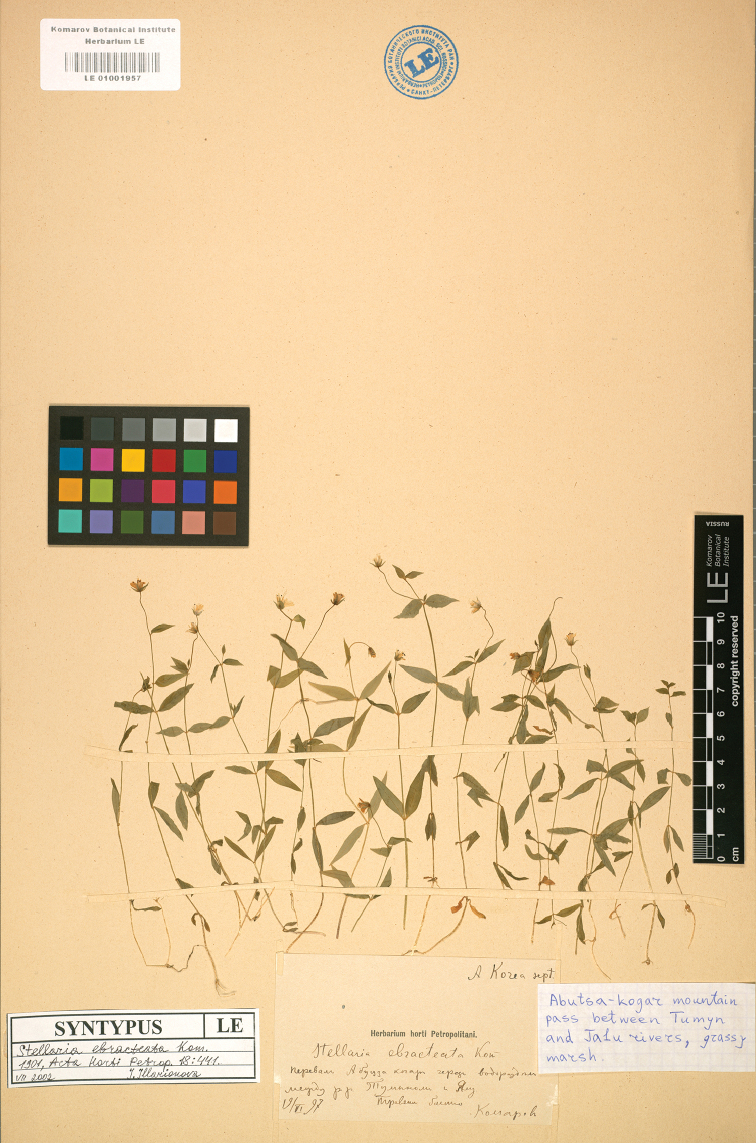
Lectotype of *S. ebracteata* Kom. (V. L. Komarov, *Komarov s.n.*, LE01001957).

(designated here):–North Korea, Ad trajectum Abuzsa-kogar divortium aquarum inter flumina Tumin et Jalu, June 19, 1897, *Komarov s.n.* (LE01001957 digital image!, Figure 3; Isolectotype: North Korea, Trajectum Czaur-ien in valle fluvii Cham-muri, *Komarov s.n.*, LE barcode LE01001956 digital image!).

#### Note.

Komarov described *S. ebracteate* and cited several specimens “*Komarov s.n.*, 18–27/VI 1894; *Komarov s.n.*, 24/V 1897; *Komarov s.n.*, 12/VI 1897; *Komarov s.n.*, 19/VI 1897” collected by himself, but never designated any one of them as holotype in the protologue. According to Stafleu and Cowan (1979), Komarov’s type specimens were deposited in LE. Two specimens traced in LE (LE01001957 digital image! and LE01001956 digital image!), match “*Komarov s.n.*, 12/VI 1897, *Komarov s.n.*, 19/VI 1897” in the protologue, and should be considered as syntypes following Arts. 9.6 and 40 Note 1 of the ICN (Turland et al. 2018). Unfortunately, due to the possible loss or destruction of specimens, the specimens “*Komarov s.n.*, 18–27/VI 1894” couldn’t be found. Two specimens traced in LE have Komarov’s script “*Stellaria ebracteate* Kom.”, the description of collecting location, and the label “SYNTYPUS”. Since LE01001957 is morphologically complete with the well presence of flower, inflorescence, and root, LE01001957 is designated here as the lectotype following Art. 9.3 and 9.4 of the ICN (Turland et al. 2018).

### Stellaria filicaulis

Taxon classificationPlantaeCaryophyllalesCaryophyllaceae

Makino, Bot. Mag. (Tokyo). 15: 113 (1901)

71F7952E-ADCA-5025-A7A4-5AE1C2811C3D

#### Lectotype

**Figure 4.**
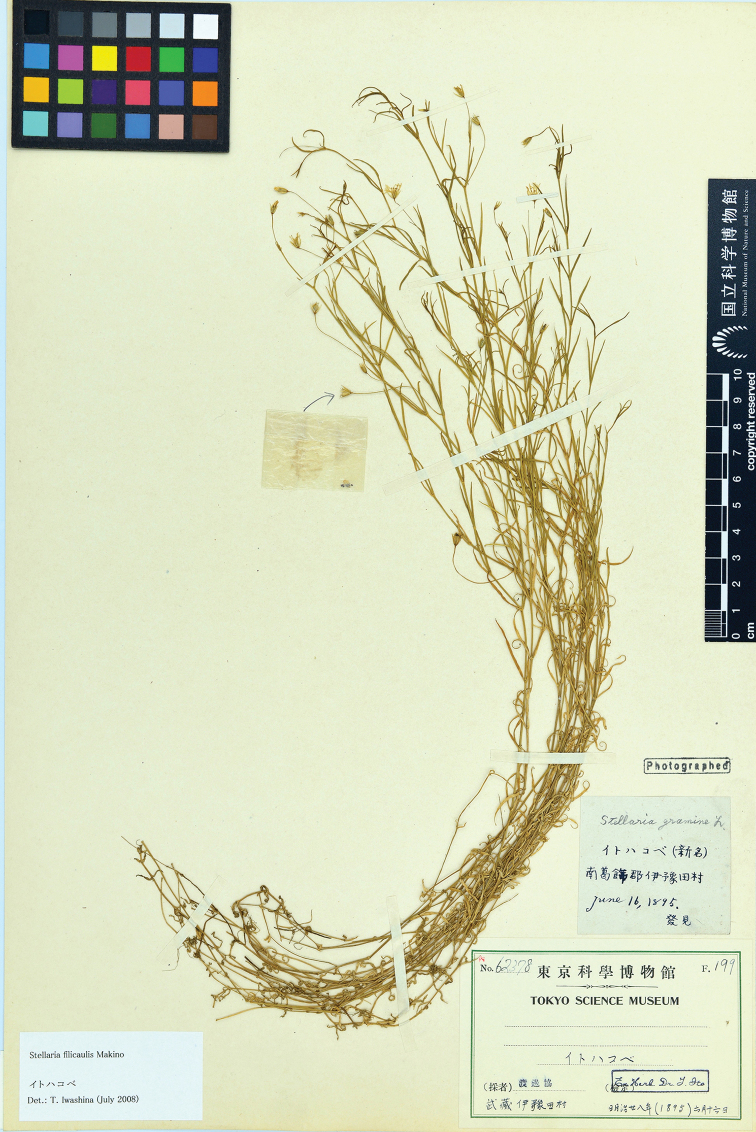
Lectotype of *S. filicaulis* Makino (Kano Watanabe, *Watanabe s.n.*, TNS62378).

(designated here):–Japan, Tokyo, Koiwa-mura, June 16, 1895, *Watanabe s.n.* (TNS62378 digital image!, Figure 4; Isolectotypes: Japan, Musashi Prov., Koiwa-mura, Yoda, *Makino s.n.*, MAK barcode MAK009391 digital images!, Japan, Hitachi Prov., Itako, *Suzuki s.n.*, MAK barcode MAK009392 digital images!, Japan, Musashi Prov., Koiwa-mura, Yoda, *Watanabe s.n.*, MAK barcode MAK010156 digital image!).

#### Note.

Makino first described *S. filicaulis* without designating a specimen as holotype but mentioned four specimens “*Watanabe s.n.*, June 16, 1895; *Makino s.n.*, June 23, 1895; *Watanabe s.n.*, June 16, 1895; *Suzuki s.n.*, May 19, 1901” in the protologue. Yet following Arts. 9.6 and 40 Note 1 of the ICN (Turland et al. 2018), these specimens should be treated as syntypes. According to Stafleu and Cowan (1981 and 1988), the original specimens were traced in GH, TI and MAK, but no specimens could be found in GH and TI mentioned in the protologue. Tropicos (Tropicos 2020) cited “Type-Protologue: *K. Watanabe s.n.* in TI”, but related specimens were not found in TI. Fortunately, original specimens in TNS (TNS62378 digital image!) and MAK (MAK009391–MAK009392 digital images!, MAK010156 digital image!) were traced, with a description of the collecting location and date agreeing with the protologue. They could be confirmed as original specimens. Moreover, Makino might have described *S. filicaulis* based on one of these specimens because it has a label containing a message which means a new name. Hence, TNS62378 is designated here as the lectotype for its good preservation, the numerous flowers and fruits, and also greatly agreeing with the protologue according to Art. 9.3 and 9.4 of the ICN (Turland et al. 2018).

### Stellaria pusilla

Taxon classificationPlantaeCaryophyllalesCaryophyllaceae

EM. Schmid, Repert. Spec. Nov. Regni Veg. 31: 41 (1932)

A5473E49-2589-5971-A374-BAA710FEE73B

#### Lectotype

**Figure 5.**
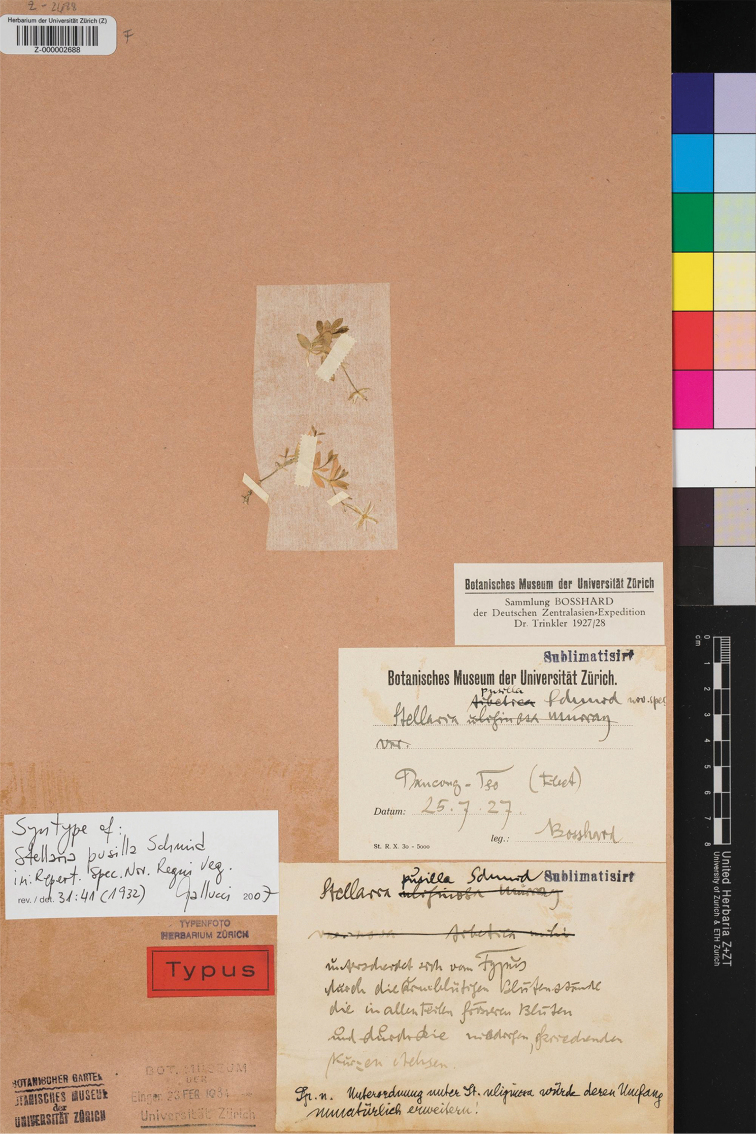
Lectotype of *S. pusilla* Em. Schmid (Walter Bosshard, *Bosshard s.n.*, Z000002688).

(designated here):–China, Tibet, Panggong Tso, July 25, 1927, *Bosshard s.n.* (Z000002688 digital image!, Figure 5; Isolectotype: China, Tibet, Panggong Tso, *Bosshard s.n.*, Z barcode Z000002689 and Z000002690 digital image!).

#### Note.

*S. pusilla* was described by Schmid based on three specimens “*Bosshard s.n.*, 25. VII. 1927; *Bosshard s.n.*, 29. VII. 1927; *Bosshard s.n.*, 13. VIII. 1927” collected by Bosshard from China, but he didn’t designate one of them as holotype in the protologue. Plants of Central Asia (Grubov and Kozhevnikov 2007) cited “Panggon Toso July 25, 1927 (typus)”. Yet following Art. 9.6 and Art. 40 Note 1 of the ICN (Turland et al. 2018), none of them can be treated as holotype, but all should be considered as syntypes. Bosshard selected type specimens that were deposited at W and Z (Stafleu and Cowan 1993). Three original specimens were traced deposited in Z (Z000002688 digital image!, Z000002689 digital image!, and Z000002690 digital image!). All of them agreed with the collection location and date in the protologue and had a label “Typus” and another label “Syntype of *Stellaria pusilla* EM. Schmid” written by Sallucn at the same time. Given its label “*Stellaria pusilla* Schmid nov. spes” and its good preservation, the presence of flower and lower part of the plant, Z000002688 is designated here as the lectotype following Art. 9.3 and 9.4 of the ICN (Turland et al. 2018).

## Supplementary Material

XML Treatment for Stellaria depressa

XML Treatment for Stellaria yunnanensis

XML Treatment for Stellaria ebracteata

XML Treatment for Stellaria filicaulis

XML Treatment for Stellaria pusilla

## References

[B1] Chen SL Rabeler RK (2001) *Stellaria* L. In: Wu ZY Raven PH Hong DY (Eds) Flora of China. Flora of China.Beijing: Science Press, & St Louis: Missouri Botanical Garden Press 26: 11–29.

[B2] Franchet AR (1886) Bulletin de la Société Botanique de France. La Société 33: 433. 10.1080/00378941.1886.10828428

[B3] Gan QL Li XW (2014) *Stellaria zhuxiensis* (Caryophyllaceae), a new species from Hubei, China. Annales Botanici Fennici 51(1–2): 22–24. 10.5735/085.051.0102

[B4] Grubov VI Kozhevnikov YP (2007) Plants of Central Asia – Plant Collection from China and Mongolia. Science Publishers, 33 pp. 10.1201/b10754

[B5] Komarov VL (1901) Trudy Imperatorskago S.-Peterburgskago Botaničeskago Sada. Imperatorskiǐ S.-Peterburgskiǐ botanicheskiǐ sad. 18: 441.

[B6] Makino T (1901) Botanical Magazine, Tokyo. Tokyo Botanical Society 15: 1–113.

[B7] Schmid E (1932) Repertorium Specierum Novarum Regni Vegetabilis. Berlin. Selbstverlag des Herausgebers 31: 1–41. 10.1002/fedr.19320310106

[B8] Song YF Li M Xu B Chen SF Chen L Xie CP (2020) *Stellaria multipartita* (Caryophyllaceae), a new species from Chongqing, China. Phytotaxa 442: 196–204. 10.11646/phytotaxa.442.3.5

[B9] Stafleu FA Cowan RS (1976) Taxonomic literature: a selective guide to botanical publications and collections with dates, commentaries and types. Bohn, Scheltema & Holkema, Utrecht. https://www.biodiversitylibrary.org/page/33120144

[B10] Stafleu FA Cowan RS (1979) Taxonomic literature: a selective guide to botanical publications and collections with dates, commentaries and types. Bohn, Scheltema & Holkema, Utrecht. https://www.biodiversitylibrary.org/item/103253

[B11] Stafleu FA Cowan RS (1981) Taxonomic literature: a selective guide to botanical publications and collections with dates, commentaries and types. Bohn, Scheltema & Holkema, Utrecht. https://www.biodiversitylibrary.org/item/104137

[B12] Stafleu FA Cowan RS (1988) Taxonomic literature: a selective guide to botanical publications and collections with dates, commentaries and types. Bohn, Scheltema & Holkema, Utrecht. https://www.biodiversitylibrary.org/item/103250

[B13] Stafleu FA Cowan RS (1993) Taxonomic literature: a selective guide to botanical publications and collections with dates, commentaries and types. Bohn, Scheltema & Holkema, Utrecht. https://www.biodiversitylibrary.org/item/103859

[B14] Tropicos (2020) Tropicos.org. Missouri Botanical Garden. http://www.tropicos.org [accessed: 25.09.2020]

[B15] Turland NJ Wiersema JH Barrie FR Greuter W Hawksworth DL Herendeen PS Knapp S Kusber WH Li DZ Marhold K May TW McNeill J Monro AM Prad J Price MJ Smith GF (2018) International Code of Nomenclature for algae, fungi, and plants (Shenzhen Code) adopted by the Nineteenth International Botanical Congress Shenzhen, China, July 2017. Regnum Vegetabile 159. Koeltz Botanical Books, Glashütten. 10.12705/Code.2018

[B16] Wang WQ Xu HF Shen KL Su ZW Ma ZH (2020) *Stellaria pentastyla* (Caryophyllaceae), a new species from Yunnan (China). Phytotaxa 435(1): 69–75. 10.11646/phytotaxa.435.1.9

[B17] Wu CY Ke P (1996) *Stellaria* L. In: Tang CL Ke P Lu DQ Zhou LH Wu CY (Eds) Flora Reipublicae Popularis Sinicae.Science Press, Beijing 26: 93–158.

[B18] Xu HF Ma ZH (2018) *Stellaria abaensis* (Caryophyllaceae), a new species from China. Phytotaxa 383(1): 1–48. 10.11646/phytotaxa.383.1.2

[B19] Xu HF Jiang YH Su ZW Ma ZH (2020) Pollen morphology of *Stellaria* (Caryophyllaceae) from China and its systematic implications. Phytotaxa 429(2): 91–119. 10.11646/phytotaxa.429.2.2

[B20] Yang F Liu XL Li RY Tian Y Wang HC (2020) *Stellaria procumbens* sp. nov. and *S. amplexicaulis* comb. & stat. nov. (Caryophyllaceae) from Southwest China. Phytotaxa 435: 192–202. 10.11646/phytotaxa.435.2.6

